# A genome-wide association study for corneal curvature identifies the platelet-derived growth factor receptor alpha gene as a quantitative trait locus for eye size in white Europeans

**Published:** 2013-01-03

**Authors:** Jeremy A. Guggenheim, George McMahon, John P. Kemp, Saeed Akhtar, Beate St Pourcain, Kate Northstone, Susan M. Ring, David M. Evans, George Davey Smith, Nicholas J. Timpson, Cathy Williams

**Affiliations:** 1 School of Optometry, Hong Kong Polytechnic University, Kowloon, Hong Kong; 2 MRC Centre for Causal Analyses in Translational Medicine, School of Social and Community Medicine, University of Bristol, Bristol, U.K; 3 Cornea Research Chair, Department of Optometry & Vision Sciences, College of Applied Medical Sciences, King Saud University, Riyadh, Saudi Arabia; 4 School of Social and Community Medicine, University of Bristol, Bristol, U.K

## Abstract

**Purpose:**

Corneal curvature is a key determinant of the refractive power of the eye. Variants in two genes, FKBP12-rapamycin complex-associated protein 1 (*FRAP1*) on chromosome 1p36.2 and platelet-derived growth factor receptor alpha (*PDGFRA*) on chromosome 4q12, have shown genome-wide significant association with normal variation in corneal curvature in a study of subjects of Asian origin. Variants at the *PDGFRA* locus have also shown genome-wide significant association with corneal astigmatism. Whether these variants influence other ocular parameters such as axial length has yet to be reported. We performed a genome-wide association study for corneal curvature in white European subjects from a population-based birth cohort, with the aim of replicating and extending the above findings.

**Methods:**

White European children participating in the Avon Longitudinal Study of Parents and Children (ALSPAC) birth cohort were examined at age about 15.5 years (95% confidence interval=15.45 to 15.48 years). Radius of corneal curvature and axial eye length were measured with an IOLmaster. DNA samples were genotyped with Illumina HumanHap550 arrays and untyped variants imputed using MACH, with CEU individuals from HapMap release 22, Phase II NCBI B36, Single Nucleotide Polymorphism database 126 as the reference panel. Association between corneal curvature and single nucleotide polymorphism (SNP) genotype was tested, genome-wide, using mach2qtl, with sex as a covariate (n=2023; 46.6% male).

**Results:**

The variant exhibiting the strongest evidence for association with corneal curvature (rs6554163; p=2.8×10^−6^) was located in the same linkage disequilibrium block as the previously discovered *PDGFRA* variants. Meta-analysis of the current and prior findings enhanced the evidence for association (rs17084051, p=4.5×10^−14^). rs6554163 genotype predicted 1.0% of variation in corneal curvature. In addition, these *PDGFRA* variants were associated with axial eye length, predicting 0.6% of the normal trait variation (p=5.3×10^−4^). Each copy of the minor allele of variants at the locus also increased the risk of corneal astigmatism in this white European cohort (odds ratio [OR]=1.24, 95% confidence interval=1.07–1.45; p=0.006).

**Conclusion:**

As in Asians, variants at the *PDGFRA* locus influence corneal curvature (and corneal astigmatism). However, rather than affecting corneal curvature in isolation, this locus influences the size of the eye while maintaining its scaling.

## Introduction

Sharp vision requires light to be focused precisely onto the photoreceptor layer of the retina by the combined action of the cornea and the crystalline lens. The cornea is the major refracting element, and its curvature must be carefully coordinated with the dimensions of the other component parts of the growing eye during childhood. A failure in this coordination leads to the refractive errors myopia and hyperopia, while asymmetry of the cornea’s curvature in different orientations, corneal astigmatism, can similarly impair vision [1]. Refractive errors are widespread: For instance, they now affect more than 40% of the population in the United States [2] and the majority of children leaving high school in Hong Kong, Singapore, and Taiwan [3,4]. Excessive corneal steepness is a hallmark feature of keratoconus, and excessive flatness of cornea plana [5,6].

Apart from the overall size of the eye, known predictors of corneal curvature include height and sex. To date, however, little is known about the genetic regulation of eye size at the molecular level, despite ocular component dimensions being heritable [7-9]. Recent genome-wide association studies (GWASs) have begun to address this question, with loci associated with corneal curvature [10], corneal astigmatism [11], refractive error [12,13], and high myopia [14-18] having been reported, mostly in subjects of Chinese ethnicity. As yet, few of these GWAS findings have been replicated independently or have already failed to replicate in subjects of the same or different ethnicity when examined in more detail [19-22]. Our aim was to replicate, in a large collection of European children, associations detected in prior studies on corneal curvature and astigmatism in Asians, which implicated the involvement of the platelet-derived growth factor receptor alpha (*PDGFRA*) gene in the determination of both traits and the FKBP12-rapamycin complex-associated protein 1 (*FRAP1*, also known as *MTOR*) gene in determining corneal curvature [10,11].

## Methods

### Avon Longitudinal Study of Parents and Children

The Avon Longitudinal Study of Parents and Children (ALSPAC) recruited 14,541 pregnant women residing in Avon, UK, with expected dates of delivery from April 1, 1991, to December 31, 1992. Of the initial 14,541 pregnancies, 13,988 children were alive at 1 year. Data have been collected through various methods including self-completion questionnaires sent to the mother, to her partner, and after age 5 to the child; direct assessments and interviews in a research clinic; and biologic samples and links to school and hospital records. The original cohort was largely representative of the UK 1991 Census: However, there was a trend for greater loss at follow-up for families of low socioeconomic status and of non-white ethnic origin [23]. Ethical approval for the study was obtained from the ALSPAC Law and Ethics committee and the three local research-ethics committees.

### Biometry

Non-invasive measurements of axial eye length and corneal curvature were performed during a visit to a research clinic when participants were aged approximately 15 years old (Zeiss IOLmaster instrument, Carl Zeiss Meditec, Welwyn Garden City, UK). Height was measured to the last complete mm using a Harpenden Stadiometer. Refractive error was assessed with non-cycloplegic autorefraction (Canon R50 instrument, Canon USA Inc., Lake Success, NY). For calculating corneal astigmatism, the difference in corneal refractive power in the steepest meridian was subtracted from that in the flattest, using the formula F=(n-1)/r, where F is corneal power in diopters (D), n is the refractive index of the cornea (1.332), and r is the corneal curvature in meters [24]. For corneal astigmatism, individuals were excluded if the level of corneal astigmatism in either eye was above an arbitrarily selected threshold of 4 D or the difference in corneal astigmatism between the two eyes was beyond 4 standard deviations (SD) from the mean (23 out of 2,617 individuals were excluded; 0.9%). For the other traits, outlier readings were identified separately for boys and girls, as measurements falling outside 4 SD from the mean (for either the measures themselves or the difference between measures in fellow eyes). For the traits height, axial length, and corneal curvature, 1 out of 2,710 (0.03%), 28 out of 2,729 (1.0%), and 35 out of 2,617 (1.3%) individuals, respectively, were excluded as outliers. Non-cycloplegic autorefraction readings were filtered to exclude outliers as described [25], resulting in the exclusion of 78 out of 4,837 individuals (1.6%). Trait values for corneal curvature, axial length, corneal astigmatism, and refractive error were averaged between fellow eyes to maximize statistical power [26]. Corneal astigmat cases were defined as subjects with an average corneal cylinder power ≥0.75 D, while corneal astigmat controls were defined those with average corneal power <0.75 D, as adopted by Fan et al. [11]. Subjects were classified as myopic if their spherical equivalent refractive error, averaged between the two eyes, was ≤–1.00 D [25].

### Genotyping, transcriptomics, and statistical analyses

DNA samples were genotyped at two different sites, the Wellcome Trust Sanger Centre, Cambridge, UK (“Sanger”) and the Laboratory Corporation of America, Burlington, North Carolina (“Labcorp”) using Illumina HumanHap 550 bead arrays. Quality control (QC) procedures were performed as described previously [27]. Briefly, individuals were excluded using the filtering thresholds: >3% missing genotypes, >10% identity-by-descent (IBD), average heterozygosity (<0.320 or >0.345 for Sanger data; <0.310 or >0.330 for LabCorp data), or sex discrepancy. Multidimensional scaling analysis was used to compare ALSPAC individuals with HapMap II, release 22, reference individuals of European, Han Chinese, Japanese, and Yoruba descent: Subjects with non-European ancestry were removed. Single nucleotide polymorphisms (SNPs) were excluded using the filter thresholds: <95% call rate, <1% minor allele frequency (MAF), Hardy–Weinberg equilibrium p value <5×10^−7^. After QC, genotypes for 464,311 autosomal SNPs were available for 8,365 individuals. Genotypes were imputed at 2,543,887 sites using MACH, with a reference panel of CEU subjects (HapMap release 22, Phase II NCBI B36, Single Nucleotide Polymorphism database 126). SNPs with a MAF >1% and an imputation quality Rsqr >0.3 were taken forward. Meta-analysis with Han et al.’s [10] previous results were carried out using the inverse variance weighting option of METAL [28].

Genome-wide gene expression in cultured Epstein-Barr virus (EBV) transformed lymphoblastoid cell lines was assessed for 997 unrelated ALSPAC participants using Illumina HumanHT-12 v3 Expression BeadChip arrays, as described previously [29]. These arrays contain >47,000 probes, two of which target PDGFRA (ILMN_1681949 and ILMN_2086470). For the 875 children who had gene expression and genotype data available, the expression level of each probe was transformed by applying an inverse normal transformation [30] and analyzed as a quantitative trait using an additive, expression quantitative trait locus (eQTL) model in mach2qtl, with sex as a covariate.

There were 2,023 subjects with data available for corneal curvature and whose genotype data passed the QC filters. A GWAS was performed for corneal curvature (transformed to a normal score, i.e., a normally distributed variable with a mean of zero and a standard deviation of one), including sex as a covariate, using an additive model in mach2qtl. There was no indication of genomic inflation (λ=1.00). Subsequent analysis of the imputed SNP rs6554163 was conducted using multiple linear regression in SPSS 18 (SPSS Inc., Chicago, IL) for 1,968 individuals who had data available for corneal curvature, axial length, height, and SNP genotype. Models were constructed for the dependent variable corneal curvature, with predictor variables sex, height, and rs6554163 genotype dosage [31]. Analogous models were constructed with axial length as the dependent variable. In all models, the trait variables corneal curvature, axial length, and height were examined as normal scores to facilitate comparisons across models. Association between the presence/absence of corneal astigmatism (or myopia) and rs6554163 genotype was assessed with the chi-square test.

### Electron microscopic immune-labeling

Human corneal tissue procurement and use were conducted in accordance with the Declaration of Helsinki and local regulations, and were approved by the Research Ethics Committee of King Saud University. Unless specified otherwise, reagents were obtained from TAAB Laboratories Equipment Ltd (Aldermaston, UK). Pieces of tissue 1 mm^2^ were ﬁxed in freshly prepared 4% paraformaldehyde in 0.1 M phosphate for 2 h at 4 °C. Tissues were processed at low temperatures and were embedded in LR White resin at −20 °C for 48 h under ultraviolet light. Ultrathin sections were collected on 200 mesh formvar-coated carbon nickel grids. A rabbit polyclonal antibody to amino acids 1035–1053 of PDGFRA (#LS-B6056, LS Bio, Seattle, WA) was used as the primary antibody, with 10-nm gold particle conjugated goat antirabbit immunoglobulin G (Biocell, Cardiff, UK) as the secondary antibody.

Sections were incubated at room temperature with phosphate buffer saline (PBS; 0.1 M sodium phosphate, 0.15 M NaCl, pH 7.4) containing 0.1% bovine serum albumin (PBS-BSA) for 15 min, PBS-BSA containing 5% normal goat serum (PBS-NGS) for 30 min, and primary antibody (1:50 to 1:500) in PBS-NGS overnight. Sections were rinsed (three washes, 5 min each) sequentially with 0.05 M Tris-buffered saline (TBS) pH 7.4, TBS containing 0.2% BSA, and TBS pH 8.4 containing 1% BSA, and then incubated in secondary antibody-immunogold conjugate (1:25 in TBS pH 8.4 containing 1% BSA) for 50 min. Unbound secondary antibody was removed by rinsing (three washes, 5 min each) in TBS-BSA, PBS, and finally distilled water. As a control, the primary antibody was omitted. Grids were stained with 2% aqueous uranyl acetate and lead citrate and examined in a Jeol 1400 transmission electron microscope (Jeol Ltd, Tokyo, Japan).

## Results

**Figure 1:**
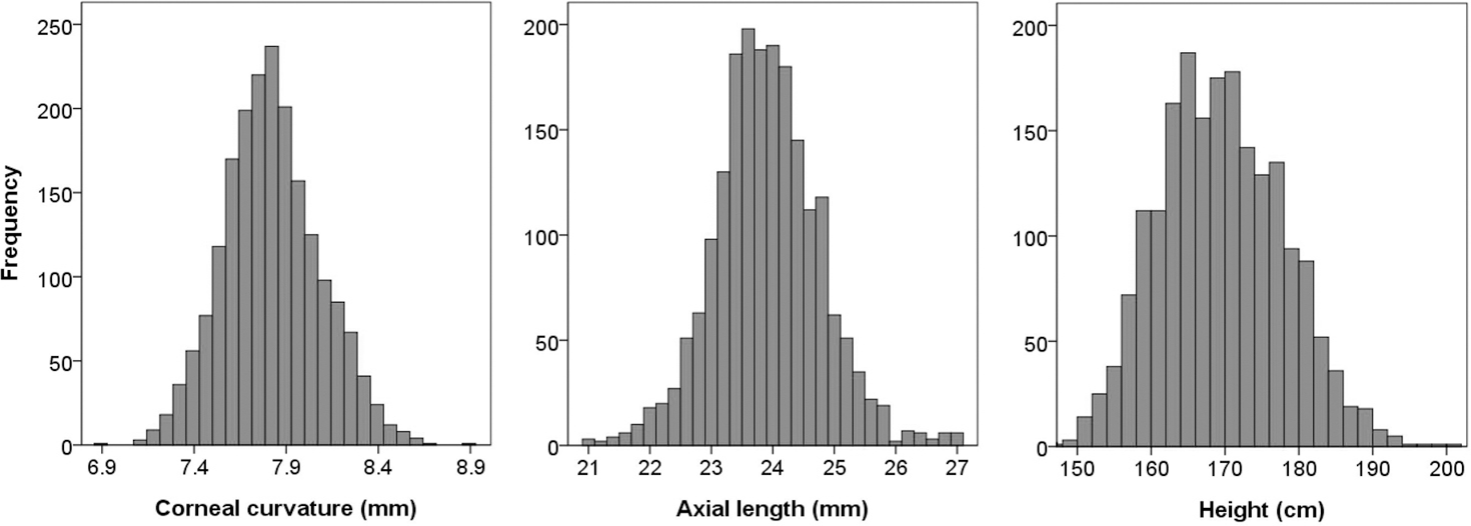
Distributions of biometric data. (n=1968 subjects with complete phenotypic and genetic information).

**Figure 2:**
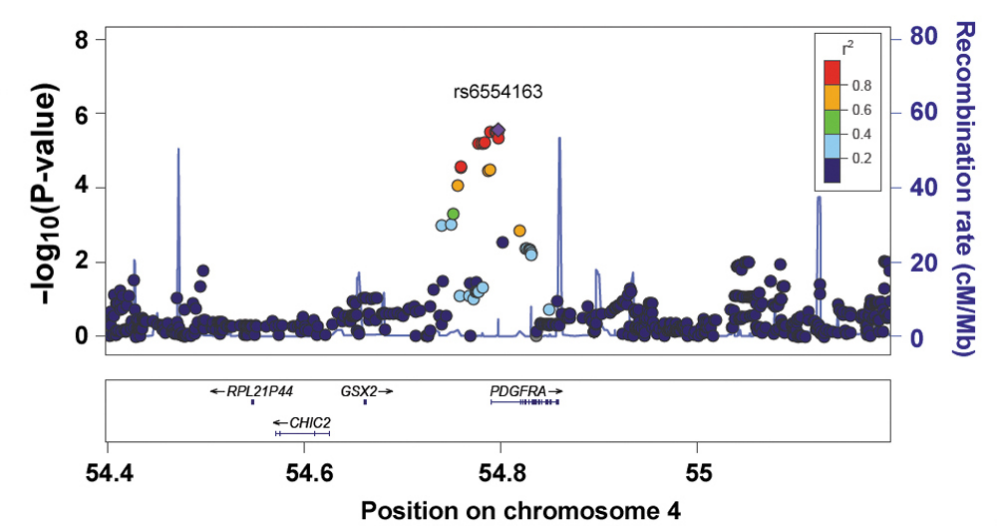
Genomic region plot from the Avon Longitudinal Study of Parents and Children genome-wide association study for corneal curvature.

Taken at an average age of 15.5 years, corneal curvature measurements in a sample of 2,023 white European children from a UK birth cohort had a Gaussian distribution and a mean value of 7.82 mm (95% confidence interval [CI]=7.81 to 7.83) and, on average, were lower (steeper) in girls than boys (Table 1 and Figure 1). A GWAS for corneal curvature did not yield any SNP markers with a p value <5×10^−8^, a value widely accepted as indicating genome-wide significance [32]. However, the SNP with the lowest p value in our GWAS (rs6554163, p=2.8×10^−6^; Table 2) was located at the 3′ end of the *PDGFRA* gene on chromosome 4 (Figure 2), which was recently identified by Han et al. [10] as one of two genes harboring genome-wide significant variants influencing corneal curvature in four Asian samples. rs6554163 resides in a region of low recombination frequency on chromosome 4 spanning approximately 0.16 Mb and that includes only the *PDGFRA* gene (Figure 2). A meta-analysis of the results from the present white European sample and the four samples recruited from Singapore by Han et al. [10], which comprised Chinese adults (SP2), Malay adults (SiMES), Indian adults (SINDI), and Chinese children (SCORM), enhanced the evidence for association observed in the initial study (combined meta-analysis including ALSPAC and Asian samples, p=4.5×10^−14^ for SNP rs17084051; **Appendix 1**). The study by Han et al. [10] identified a second genome-wide significant locus associated with corneal curvature in Asians, centered on the *FRAP1* gene at chromosome 1p36.2. However, there was no evidence of replication for this locus in our sample of white European children (all p>0.05; **Appendix 1**).

**Table 1:** Biometric data for ALSPAC subjects. (n=1968 subjects with complete information available: corneal curvature, axial length, height and genotypes).

Parameters	Boys (n=914)		Girls (n=1054)	
	mean	95% CI	mean	95% CI
Age (years)	15.44	15.42-15.46	15.47	15.45-15.49
Height (cm)	174.8	174.3-175.3	164.7	164.3-165.0
Axial length (mm)	23.7	23.64-23.75	23.16	23.11-23.21
Corneal curvature (mm)	7.886	7.869-7.903	7.766	7.751-7.781

**Table 2:** ALSPAC GWAS results for the PDGFRA region. (n=2023 subjects with corneal curvature and genetic information available).

Marker	CHR	POS	A1	Freq	Beta	SE	P value
rs6554163	4	54797316	A	0.22	−0.176	0.037	2.78×10^–6^
rs7678144	4	54797182	C	0.22	−0.175	0.037	2.81×10^–6^
rs4864862	4	54795246	A	0.22	−0.175	0.037	2.99×10^–6^
rs4864863	4	54795588	G	0.22	−0.175	0.037	2.99×10^–6^
rs6850748	4	54793921	G	0.22	−0.174	0.037	3.06×10^–6^
rs1800812	4	54789386	T	0.22	−0.174	0.037	3.06×10^–6^
rs6836215	4	54797498	C	0.21	−0.177	0.039	4.43×10^–6^
rs7673984	4	54783518	T	0.22	−0.168	0.037	6.04×10^–6^
rs11133315	4	54776915	A	0.22	−0.168	0.037	6.20×10^–6^
rs7682912	4	54780377	G	0.22	−0.168	0.037	6.20×10^–6^

CHR=Chromosome; POS=human genomic position in reference assembly genome build 36.3; A1=test allele; Freq=frequency of test allele; Beta=standardised regression coefficient per copy of test allele; SE=standard error of beta.

**Figure 3:**
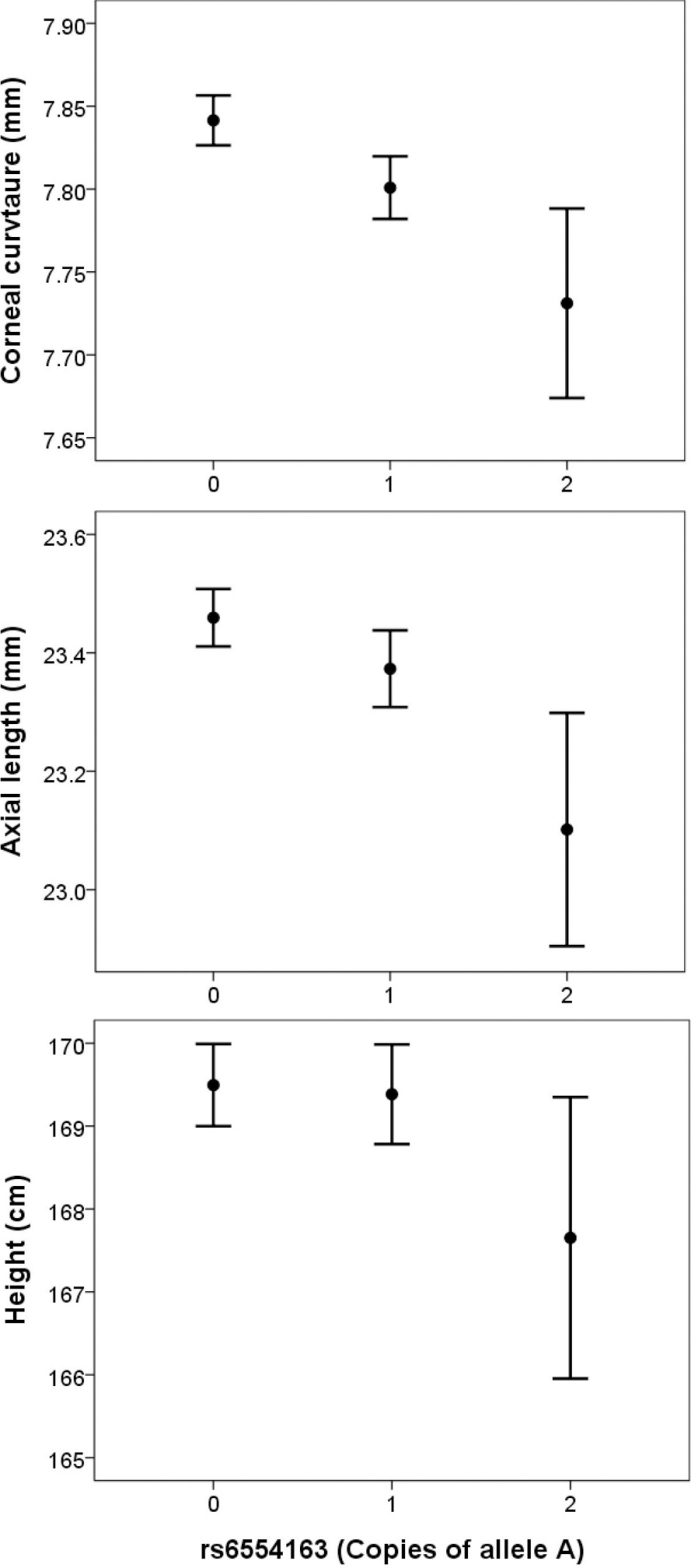
Associations between genotype at single nucleotide polymorphism rs6554163 and corneal curvature, axial length, and height in 15-year-old children (n=914 boys and 1054 girls) from the Avon Longitudinal Study of Parents and Children cohort. Error bars show 95% confidence interval.

Han et al.’s [10] findings were consistent with a single causal variant being responsible for the association between SNP genotypes in the *PDGFRA* region and corneal curvature. However, GWAS results alone cannot distinguish from among a set of associated SNPs which (if any) is the causal variant. Nevertheless, because of the high linkage disequilibrium (LD) and similar allele frequencies of the associated SNPs, each of the SNPs in the associated *PDGFRA* region can be regarded as a surrogate for the (presumed single) causal variant. In support of this reasoning, including the imputed genotype dosage of SNP rs6554163 as a covariate in the corneal curvature GWAS completely removed evidence of association with other variants in the region (**Appendix 1**). We used linear regression models to evaluate the extent the lead SNP rs6554163 in the *PDGFRA* gene influenced not just corneal curvature but also the potentially related traits, axial eye length and body height. Under an additive model of SNP effects, rs6554163 retained an association with corneal curvature of equivalent magnitude before and after controlling for height (Table 3): Standardized beta coefficient before=–0.167 (95% CI=−0.093 to −0.241) and after=–0.163 (95% CI=−0.091 to −0.235). In both models, rs6554163 predicted 1.0% of the variation in corneal curvature. This SNP was also found to exert a comparable influence on axial length, which again was unaffected by statistically controlling for height (Table 4): Standardized beta coefficient before=–0.128 (95% CI=−0.056 to −0.200) and after=–0.125 (95% CI=−0.054 to −0.195). In both cases, rs6554163 predicted 0.6% of the variation in axial length. There was little evidence of association between the rs6554163 genotype and height itself: Standardized beta coefficient=–0.012 (95% CI=−0.049 to 0.074; p=0.69). The average effect of the *PDGFRA* gene variant on corneal curvature, axial length, and height is presented in Figure 3.

**Table 3:** Linear regression model for dependent variable corneal curvature with predictors sex, genotype at rs6554163 under an additive model, with and without height (n=1968).

A – Including height in model				
Parameter	Beta	SE	P value	Partial η^2^
sex	0.136	0.053	9.98×10^–3^	0.003
height (normal score)	0.265	0.026	1.96×10^–23^	0.049
rs6554163	−0.163	0.037	9.19×10^–6^	0.010

B – Omitting height from the model				
Parameter	Beta	SE	P value	Partial η^2^
sex	0.447	0.044	8.30×10^–24^	0.050
rs6554163	−0.167	0.038	1.02×10^–5^	0.010

All variables were transformed to normal scores for analysis.

**Table 4:** Linear regression model for dependent variable axial length with predictors sex, genotype at rs6554163 under an additive model, with and without height (n=1968).

**A – Including height in model**				
Parameter	Beta	SE	P value	Partial η^2^
sex	0.339	0.052	5.86×10^–11^	0.022
height (normal score)	0.243	0.026	8.66×10^–21^	0.044
rs6554163	−0.125	0.036	5.34×10^–4^	0.006

**B – Omitting height from the model**				
Parameter	Beta	SE	P value	Partial η^2^
sex	0.624	0.043	7.61×10^–46^	0.098
rs6554163	−0.128	0.037	3.48×10^–4^	0.006

All variables were transformed to normal scores for analysis.

Separate from Han et al.’s [10] work, a GWAS performed by Fan et al. [11] demonstrated that variants in the same region of the *PDGFRA* gene that influenced corneal curvature also influenced the risk of corneal astigmatism. Fan et al. [11] surveyed five Asian cohorts recruited from Singapore, namely, the four cohorts listed above in the relation to Han et al.’s [10] work, plus a sample of Chinese parent-infant trios (STARS). For the lead SNP in that study (rs7677751, MAF 0.19 to 0.26), each copy of the minor allele was associated with an increased risk of individuals being classified as a corneal astigmat (OR=1.26, 95% CI=1.16 to 1.36, in the meta-analysis of five studies). In our white European subjects, SNP rs6554163 located in the *PDGFRA* high LD region was associated with the risk of corneal astigmatism to a similar degree and in the same direction as that observed in the Asian subjects studied by Fan et al. [11]. Specifically, each copy of the minor allele of rs6554163 was associated with an increased risk (OR) of corneal astigmatism of 1.24 (95% CI=1.07 to 1.45; p=0.006) in the ALSPAC subjects (Table 5). In contrast to these results for corneal astigmatism, the rs6554163 genotype did not predict an individual being classified as myopic versus non-myopic (Table 5): Each copy of the minor allele was associated with an OR for myopia of 0.98 (95% CI=0.79 to 1.20; p=0.78). In these latter analyses, sex was associated with corneal astigmatism (being more frequent in girls than boys; OR=1.406, 95% CI=1.174 to 1.683; p<0.001) but was not associated with myopia (Table 5).

**Table 5:** Binary logistic regression model for dependent variable corneal astigmatism case versus control status or myopia case versus control status, with predictors sex and genotype at rs6554163 under an additive model (n=1968).

A - Corneal astigmatism case versus control status					
Parameter	B	SE	P value	OR	95% CI
sex	0.341	0.092	2.06×10^–4^	1.406	(1.174 to 1.683)
rs6554163	0.210	0.078	0.007	1.234	(1.058 to 1.439)

B - Myopia case versus control status					
Parameter	B	SE	P value	OR	95% CI
sex	0.204	0.124	0.101	1.226	(0.961 to 1.565)
rs6554163	−0.030	0.106	0.780	0.971	(0.789 to 1.195)

To investigate whether variants at the *PDGFRA* locus contribute in *cis* to inter-individual variation in *PDGFRA* gene expression, we performed eQTL (expression quantitative trait locus) GWAS analyses. Whole genome gene expression (transcriptome) array results for EBV-transformed lymphoblastoid cell lines and high-density genotype data were available for 875 of the children from the ALSPAC birth cohort. However, for each of the two probes used to monitor *PDGFRA* expression on the transcriptome array, there was no evidence of a major eQTL on chromosome 4 in the vicinity of the *PDGFRA* gene (**Appendix 1**). This suggests that (at least in these cell lines) variants at the *PDGFRA* locus do not act as *cis* regulatory factors that influence the gene’s expression level.

**Figure 4:**
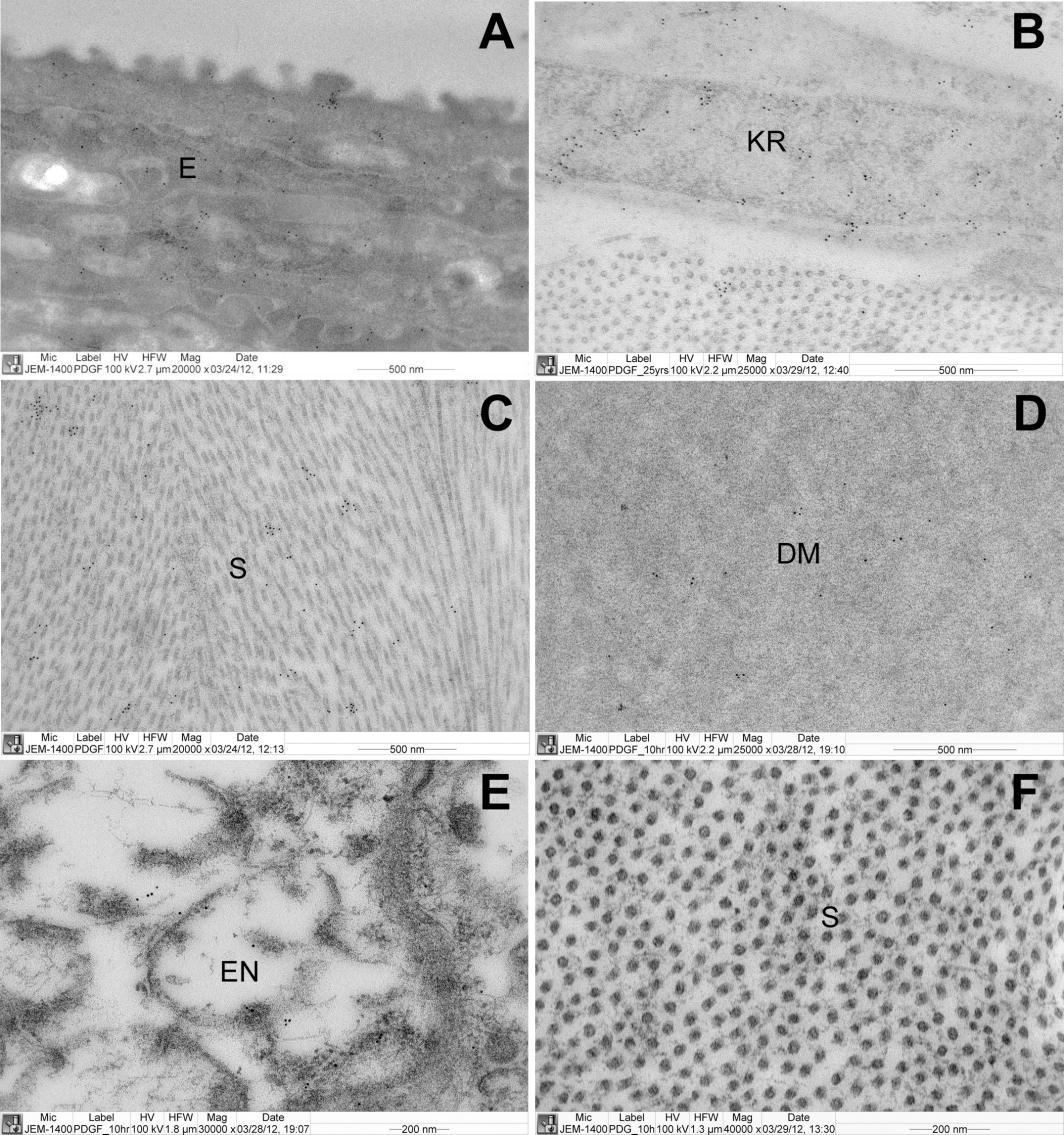
Electron microscopy antibody-labeling of platelet-derived growth factor receptor alpha in a normal human cornea. Strong labeling was seen in all layers of the epithelium (**A**). Labeling was also evident in the stromal keratocytes, especially their cell membranes (**B**) and in the surrounding extracellular matrix (**C**). Descemet’s membrane (**D**) and the endothelial monolayer (**E**) exhibited weaker labeling. No labeling was observed in control sections in which the primary antibody was omitted (**F**). Abbreviations: DM=Descemet’s membrane, E=Epithelium, EN=Endothelium, KR=Keratocyte, S=Stroma.

An antibody to PDGFRA was used to assess the protein’s ultrastructural distribution in the normal human cornea. Antibody labeling was strongest in the epithelium, all layers of which were stained, but especially the superficial layer of squamous cells (Figure 4A). Labeling was less abundant in the corneal stroma, but was present in the keratocyte cells and their surrounding extracellular matrix (Figure 4B–C). The endothelial layer and Descemet’s membrane exhibited weaker labeling (Figure 4D–E). Negative control sections showed no background labeling (Figure 4F).

## Discussion

Our finding of a strong association between variants in the *PDGFRA* gene and corneal curvature, with an effect of equivalent size and direction to that observed by Han et al. [10], suggests that *PDGFRA* variants influence corneal curvature in both Asians and white Europeans. Likewise, our finding that these same variants were associated with corneal astigmatism in the ALSPAC subjects, and that the size and direction of effect matched those reported by Fan et al. [11], implies that these *PDGFRA* variants influence corneal astigmatism in Asians and white Europeans, as well. By contrast, the lack of replication for the *FRAP1* gene locus in our sample of white European children suggests that the *FRAP1* variants influencing corneal curvature in Asians [10] are not present in Europeans, or are not well tagged by common SNPs in Europeans. Notably, patterns of LD in this region are markedly different between European (CEU) and Asian (CHB+JPT) HapMap individuals (**Appendix 1**). While our article was under review, Mishra et al. [33] also reported that *PDGFRA* variants (but not *FRAP1* variants) influenced corneal curvature in a sample of Australian subjects of European ancestry (1,788 twins and their families, plus 1,013 members of a birth cohort). These authors did not examine the effects of *PDGRFA* variants on corneal astigmatism or axial length.

Previous phenotype-correlation analyses in mice [34] and chickens [35-37] support the existence of three types of genetic “effects” that act together to determine the size of the component parts of the vertebrate eye (or, more precisely, that proportion under genetic control). These are (i) genetic effects that allometrically scale the size of the eye and body, (ii) effects that purely govern eye size, and (iii) effects restricted to determining the dimensions of each individual ocular component. The *PDGFRA* variants studied here have the characteristics of genetic effects in the second group, since (as judged by the inheritance of copies of the minor A allele of SNPs such as rs6554163; **Figure 3**) they act to reduce corneal curvature (i.e., steepen it) and to reduce axial eye length. As visual function is exquisitely dependent on the careful scaling of the eye’s component parts, observing such pleiotropy is not surprising. In terms of identifying genetic variants that influence susceptibility to disorders such as refractive error, variants that affect the expression level or function of *PDGFRA* would not seem likely candidates given this pleiotropic scaling role. Indeed, subjects’ genotypes for SNP rs6554163 were not associated with the presence/absence of myopia in ALSPAC subjects. Thus, *PDGFRA* variants appear to exert a risk of corneal astigmatism but not spherical refractive error.

Corneal curvature tends to increase (flatten) markedly during the first few years of life and then remain relatively stable during the remainder of childhood and early adulthood [38-40]. Thus, the association between corneal curvature and *PDGFRA* variants identified in the 15-year-old children examined here is likely to persist as they age, consistent with the associations previously observed in child and adult Asian populations [10]. However, axial length follows a different pattern of post-natal growth to that of corneal curvature, generally continuing to increase throughout childhood and even into early adulthood [38-40]. This suggests that although these traits share common genetic determinants, there may be differences in the age and/or duration over which these variants exert their effects. It will be of interest to explore further how the association of *PDGFRA* and axial length alters over the lifecourse, in would-be emmetropes and myopes.

Situated on chromosome 4q12, *PDGFRA* encodes the alpha isoform of a transmembrane tyrosine kinase receptor for members of the PDGF family. The gene is very widely expressed and has important roles in development and cell proliferation. We observed PDGFRA antibody labeling in all of the major cell types of the normal human cornea, as reported previously [41]. Protein levels appeared highest in the epithelium, followed by stromal keratocytes, and lower still in the endothelium. Antibody labeling was not restricted to the cell membrane in any of the three cell types, consistent [42] with the presence of intracellular stores of PDGFRA, which would permit mobilization to the cell surface in response to external stimuli. Variants in *PDGFRA* have previously been associated with susceptibility to several cancer types, certain developmental anomalies (see Genetic Association), and several red blood cell quantitative traits [43]. Pertinent to the association with corneal curvature and axial length, members of the PDGF family are known to modulate multiple aspects of extracellular matrix biology [44-48]. However, the specific physiologic mechanism through which *PDGFRA* gene variation exerts an effect on eye size awaits elucidation.

## Appendix 1.

Supporting Information. To access the data, click or select the words “Appendix 1.” This will initiate the download of a compressed (pdf) archive that contains the file.
